# 77. Risk of Virologic Failure Among Treatment-experienced Suppressed People with HIV (PWH) Treated with Single-Tablet 2-Drug (2DR) vs 3-Drug (3DR) Regimens

**DOI:** 10.1093/ofid/ofab466.077

**Published:** 2021-12-04

**Authors:** Paul Sax, Joseph J Eron, Janna Radtchenko, Helena Diaz-Cuervo, Mark Moore, Steven Santiago, Moti Ramgopal, Karam Mounzer, Richard Elion

**Affiliations:** 1 Brigham and Women’s Hospital, Boston, MA; 2 University of North Carolina at Chapel Hill, Chapel Hill, North Carolina; 3 Trio Health, Louisville, Colorado; 4 Gilead Sciences, Madrid, Madrid, Spain; 5 CareResource, Miami, Florida; 6 Midway Specialty Care Centers, Fort Pierce, Florida; 7 Philadelphia FIGHT, Philadelphia, PA; 8 George Washington University School of Medicine, Washington, DC

## Abstract

**Background:**

The single-tablet regimen (STR) dolutegravir/rilpivirine (DTG/RPV) was approved in 2017 for use in virologically suppressed PWH, followed by approval in 2019 of dolutegravir/lamivudine (DTG/3TC). There is a need to evaluate outcomes of 2DR regimens in stable switch population by measuring risk of virologic failure.

**Methods:**

This retrospective observational study of the Trio Health HIV Research Network cohort of nearly 60,000 PWH evaluated risk of virologic failure among treatment-experienced virologically suppressed PWH switching to a 2DR or 3DR STR (index = time of switch). Eligible patients (pts) were ≥ 18 yrs, treatment-experienced, suppressed at index, with post-index viral loads, ≥ 6 mo pre-index history, with dispenses for DTG/RPV, DTG/3TC or commonly used 3DR STRs initiated after Nov. 2017. Univariate comparisons were performed using *Χ*^2^ for categorical and t-test for continuous variables; Kaplan-Meier analysis was used to evaluate time to virologic failure and Cox Proportional Hazards analysis was used to evaluate risk of virologic failure accounting for age, gender, race, and baseline eGFR. Virologic failure was defined as 2 consecutive viral loads >200 cells/ml.

**Results:**

Of 1668 pts, 132 (8%) received 2DR. Significant differences in baseline characteristics between groups are shown in Table 1. In unadjusted analysis the difference in proportion of pts with virologic failure did not reach significance: 7% (9 pts) on 2DR experienced virologic failure vs 4% (65 pts) on 3DR (p=0.166). Additionally, unadjusted risk of virologic failure and time to virologic failure were not statistically different [Figure 1, Table 2].

Based on prespecified adjusted analysis accounting for race, gender, age, and baseline eGFR, the risk of virologic failure was higher for 2DR compared to 3DR STRs (HR=2.2 CI 95% 1.1-4.5, p=0.032) [Table 2]. Adjusting for baseline CD4 was not feasible due to high proportion of missing CD4 at index.

Table 1 Baseline (index) characteristics

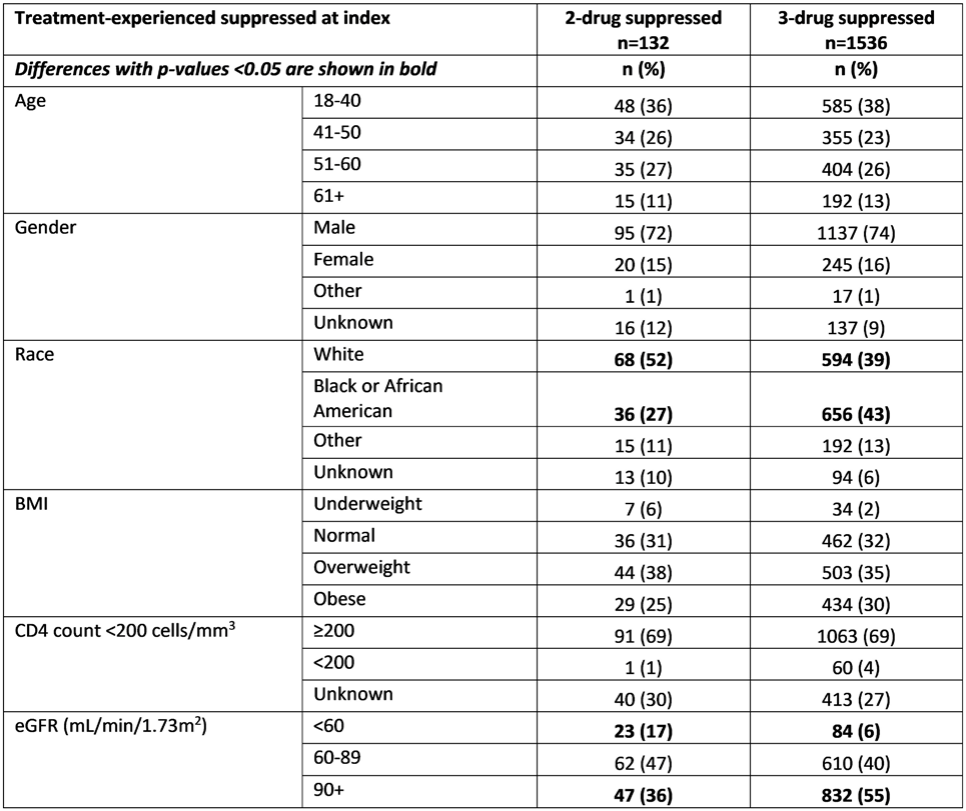

Figure 1 Unadjusted time to virologic failure

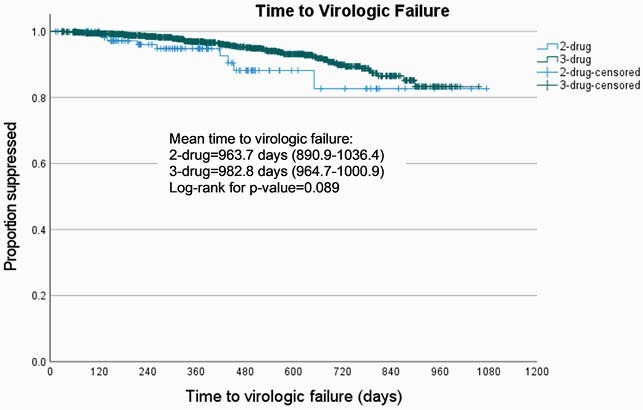

Table 2 Risk of virologic failure

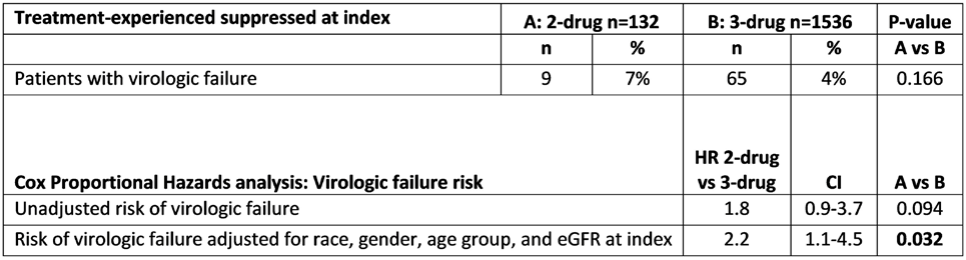

**Conclusion:**

This early evaluation showed higher risk of virologic failure among virologically suppressed pts who switched to 2DR vs 3DR STRs after adjusting for differences in baseline characteristics. Future analysis is warranted using a larger sample of 2DR pts with additional adjustment for prior regimen failure.

**Disclosures:**

**Paul Sax, MD**, **Gilead Sciences** (Consultant, Grant/Research Support)**Janssen** (Consultant)**Merck** (Consultant, Research Grant or Support)**ViiV** (Consultant, Research Grant or Support) **Joseph J. Eron, MD**, **Gilead Sciences** (Consultant, Research Grant or Support)**Janssen** (Consultant, Research Grant or Support)**Merck** (Consultant)**ViiV** (Consultant, Research Grant or Support) **Janna Radtchenko, MBA**, **Trio Health** (Employee) **Helena Diaz-Cuervo, PhD**, **Gilead Sciences** (Employee) **Mark Moore, PhD**, **Gilead Sciences** (Employee) **Steven Santiago, MD**, **Gilead Sciences** (Advisor or Review Panel member, Speaker's Bureau)**Janssen** (Speaker's Bureau) **Moti Ramgopal, MD FIDSA**, **Abbvie** (Scientific Research Study Investigator, Speaker's Bureau)**Gilead** (Consultant, Scientific Research Study Investigator, Speaker's Bureau)**Janssen** (Consultant, Scientific Research Study Investigator, Research Grant or Support, Speaker's Bureau)**Merck** (Consultant, Scientific Research Study Investigator)**ViiV** (Consultant, Scientific Research Study Investigator, Speaker's Bureau) **Karam Mounzer, MD**, **Epividian** (Advisor or Review Panel member)**Gilead Sciences Inc.** (Consultant, Scientific Research Study Investigator, Research Grant or Support, Speaker’s Bureau)**Janssen** (Consultant, Research Grant or Support, Speaker's Bureau)**Merck** (Research Grant or Support, Speaker's Bureau)**ViiV Healthcare** (Consultant, Speaker's Bureau) **Richard Elion, MD**, **Gilead Sciences** (Grant/Research Support, Advisor or Review Panel member, Speaker's Bureau)**Janssen** (Speaker's Bureau)**Proteus** (Grant/Research Support)**Trio Health** (Employee)**ViiV** (Advisor or Review Panel member)

